# 209 A pilot rehabilitation programme for individuals experiencing symptoms of long-COVID: Results from a service evaluation

**DOI:** 10.1093/eurpub/ckae114.144

**Published:** 2024-09-26

**Authors:** Jason Wilson, Kim Kensett, Rachel O’Reilly, Mark Tully

**Affiliations:** School of Sport, Ulster University, Derry/Londonderry, United Kingdom; Active Belfast, Belfast Health Development Unit, Public Health Agency, Belfast, United Kingdom; South Eastern Health and Social Care Trust, Lisburn, United Kingdom; School of Medicine, Ulster University, Derry/Londonderry, United Kingdom

## Abstract

**Purpose:**

In the wake of the COVID-19 pandemic, long-COVID has emerged as a public health issue. There are unique challenges when it comes to exercising for those with long-COVID. Therefore, this service evaluation aimed to highlight the impact of a 12-week physical activity instructor-led programme, delivered via video-conferencing, on mental wellbeing and physical function in individuals experiencing symptoms of long-COVID.

**Methods:**

Thirty-three adults with long COVID, who had previously completed a physiotherapy-led rehabilitation programme, were invited to take part in a 12-week physical activity instructor-led pilot rehabilitation programme between June 2021 and August 2022. The intervention was delivered virtually, twice weekly, by two qualified physical activity instructors. Activities included strength and balance work, core-strengthening, and functional movement exercises. Outcome measures were collected at baseline, 6 weeks and 12 weeks (post-intervention), including self-reported mental wellbeing and physical function (i.e. 2-minute walk test/2MWT, 1-minute sit-to-stand test/1MSTST and handgrip dynamometry). Due to the exploratory nature of the intervention, minimal clinically important differences (MCID) were used to determine the intervention’s impact.

**Results:**

Twenty participants completed the full intervention (i.e. 39.4% attrition rate). On average, mental-wellbeing scores increased by 2.05±2.70 units at post-intervention compared to baseline, which is within the estimated MCID of 1-3 units. In terms of the 1MSTST, this increased by 1.25±3.45 repetitions at post-intervention compared to baseline, although did not exceed the estimated MCID of at least 3 repetitions. Distance covered in the 2MWT increased by 5.96±17.42 metres at post-intervention compared to baseline, which exceeded the estimated MCID of at least 5.50 metres. Although dominant handgrip strength increased by 3.58±6.71 kilograms at post-intervention compared to baseline, this did not exceed the estimated MCID of at least 5.00 kilograms.

**Conclusions:**

A virtually delivered 12-week pilot rehabilitation programme resulted in improved mental wellbeing and aerobic capacity, in comparison to MCIDs established in other clinical populations, in individuals experiencing symptoms of long-COVID. The findings demonstrate that virtually delivered exercise interventions specifically designed for those with symptoms of long-COVID, using activities which require little-to-no equipment, may result in clinically meaningful health benefits.

**Support/Funding Source:**

This study received funding from the Belfast Health Development Unit.

